# The impact of sofosbuvir/velpatasvir/voxilaprevir treatment on serum hyperglycemia in hepatitis C virus infections: a systematic review and meta-analysis

**DOI:** 10.1080/07853890.2023.2168745

**Published:** 2023-01-19

**Authors:** Hsuan-Yu Hung, Hui-Hsiung Lai, Hui-Chuan Lin, Chung-Yu Chen

**Affiliations:** a Department of Pharmacy, Ditmanson Medical Foundation Chia-Yi Christian Hospital, Chiayi, Taiwan; b School of Pharmacy, College of Pharmacy, Kaohsiung Medical University, Kaohsiung, Taiwan; c Master Program in Clinical Pharmacy, School of Pharmacy, Kaohsiung Medical University, Kaohsiung, Taiwan; d Department of Pharmacy, Kaohsiung Medical University Hospital, Kaohsiung, Taiwan; e Department of Medical Research, Kaohsiung Medical University Hospital, Kaohsiung, Taiwan

**Keywords:** Hyperglycemia, SOF/VEL/VOX, Hepatitis C, Diabetes

## Abstract

**Background:**

The combination of Sofosbuvir (SOF), velpatasvir (VEL), and voxilaprevir (VOX) is an effective, safe rescue therapy for patients with previous treatment failure. Direct-acting antiviral (DAA) treatment for hepatitis C virus (HCV) infection in diabetics with a history of hypoglycemia could improve insulin resistance due to HCV clearance. However, some studies have shown that SOF/VEL/VOX causes grade 3 hyperglycemia and other adverse events, which contradicts the findings of other DAA studies.

**Aim:**

To analyze the incidence of grade 3 hyperglycemia of SOF/VEL/VOX for chronic HCV infection.

**Methods:**

We searched electronic databases from the inception of each database until October 2021. A random-effects model was employed to pool data. The study was conducted according to the PRISMA guidelines, and quality assessment was performed by using the Cochrane risk-of-bias tool for randomized controlled trials (RCTs). The study protocol was registered on the INPLASY database (Registration No. 2021120109).

**Results:**

Five RCTs were included in this review. Overall, 49 of 2315 patients had grade 3 hyperglycemia with a risk ratio of 0.015 (95% confidence interval, 0.010–0.020; *p* < .001), and the incidence risk ratio (IRR) for cirrhosis compared to without cirrhosis was 12.000 (95% confidence interval: 0.727–198.160), the HCV genotype 3–genotype 1 IRR was 4.13 (95% confidence interval: 1.52–11.22) in subgroup analysis. No significant differences were found within the other subgroups, in prior DAA treatment experience, and in treatment duration.

**Conclusion:**

Although the incidence of hyperglycemia was rare in diabetic patients with HCV, it is recommended that glucose levels be closely monitored during the first 3 months of therapy and that diabetes medication be modified if necessary.

## 1. Introduction

Sofosbuvir/velpatasvir/voxilaprevir (SOF/VEL/VOX) is the first once-daily orally administered regimen approved for patients with hepatitis C virus (HCV) genotypes (GTs) 1–6 in whom direct-acting antiviral (DAA) therapy has previously failed [1]. Approximately 5% of HCV patients treated with DAAs experience virologic failure [2].

The fixed-dose combination of SOF (a nucleotide analog HCV NS5B polymerase inhibitor), VEL (an HCV NS5A inhibitor), and VOX (an HCV NS3–NS4A protease inhibitor) is a safe and effective regimen to achieve sustained virologic response (SVR) across all HCV GTs in treatment-naïve (8 weeks’ duration) or treatment-experienced (12 weeks’ duration) patients with previous DAA treatment failure, regardless of their cirrhosis status [1,3]. Moreover, the tolerability of this regimen in DAA-experienced patients has been reported to be similar to that observed in DAA-naive patients [4]. This has important implications for the selection of the best retreatment strategies for these patients [3].

Epidemiological studies have demonstrated a bidirectional association between HCV and diabetes. That is to say, HCV infection triggers diabetes, and type 2 diabetes (T2DM) serves as a predisposing factor for HCV infection; besides, diabetes deteriorates HCV outcomes, thus increasing the risk for cirrhosis and hepatocellular carcinoma [5]. The exact mechanism whereby HCV infections affect glucose metabolism has not been completely understood; however, several mechanisms have been proposed, including direct viral effects, insulin resistance (IR), proinflammatory cytokines, chemokines, and other immune-mediated mechanisms [6].

Conversely, a number of studies have suggested that there is a link between DAAs and the development of hypoglycemia [7,8]. Furthermore, improved glucose metabolism and disease control have been observed in HCV-positive patients after successful clearance of HCV [9–12]. Significant reductions in fasting blood glucose values [13–15], hemoglobin (Hb) A1C levels [7,16], IR [7,11,17,18], and insulin plasma concentrations have also been reported [19]. This has helped reduce patients’ dependence on hypoglycemic medications [6,9,20,21].

Furthermore, in December 2018, Health Canada published information about the potential risk of abnormal blood glucose levels with the use of DAAs and announced that some diabetic patients initiating DAA treatment for HCV infection had experienced hypoglycemia [22]. It has been recommended that during DAA therapy for HCV, particularly within the first 3 months of care, glucose levels be closely monitored in diabetics and that their diabetes medication or doses be modified when necessary [23].

However, some studies have reported that SOF/VEL/VOX causes grade 3 laboratory abnormalities (hyperglycemia) [3,24], which is in contradiction with the findings of other DAA studies. No relevant articles on SOF/VEL/VOX have been published. The present review was conducted to pool the incidence of grade 3 hyperglycemia in randomized controlled trials (RCTs), assess possible risk factors and incidences, and provide clinicians with a reference for selecting and modifying medications during treatment.

## 2. Methods

### 2.1. Study design

This study was performed according to the Preferred Reporting Items for Systematic Reviews and Meta-Analyses (PRISMA) guidelines [25]. Additionally, the study protocol was registered on the INPLASY database (Registration No. 2021120109).

### 2.2. Literature search strategy

We conducted a comprehensive literature search of PubMed, Cochrane Library, ClinicalKey, Embase, and MEDLINE electronic databases from their inception to 12 October 2021, by using Boolean operators and search terms without language or publication year restrictions. The search was not restricted to the published English-language articles and articles that were obtained by filtering RCTs and human subjects. Details of the search strategy used for each database are available in Supplementary Table S1.

### 2.3. Outcome measures

The safety outcomes for HCV patients treated with SOF/VEL/VOX were evaluated by the rate of drug-related laboratory abnormalities. All adverse events and laboratory values were graded according to a standardized scale and the Medical Dictionary for Regulatory Activities (MedDRA, version 24.1) [26].

The primary outcome was grade 3 hyperglycemia, defined as serum glucose levels greater than 250 mg/dL. The secondary outcome was defined as grade 3 laboratory abnormalities.

### 2.4. Selection criteria

Retrieved articles were included in the review if: (1) they described SVR12 and relapse states after treatment with SOF/VEL/VOX for HCV infection; (2) the safety outcomes recorded consisted of grade 3 serum glucose parameters; and (3) data on other grade 3 laboratory abnormalities (adverse events) were present. Study eligibility was based on the initial screening of titles, methodologies, and abstracts, followed by full-text reviews.

Studies were excluded if they: lacked data on SVR12 following SOF/VEL/VOX therapy for HCV infection, or failed to measure grade 3 glucose parameters; (2) involved patients with comorbidities, human immunodeficiency virus (HIV) infection, hepatitis B infection, or any cause of liver disease other than chronic HCV infection; (3) included posttransplant patients; (4) and were book chapters, abstract-only articles, conference papers, reviews, theses, posters, editorials, and letters.

### 2.5. Data extraction

The following data were extracted from eligible studies: (1) baseline characteristics of enrolled participants: Intervention, duration, age, gender, race, HCV genotype, cirrhosis; (2) inclusion criteria for study design, efficacy outcomes including SVR and virologic relapse; (3) hyperglycemia events and drug-related laboratory abnormalities: hemoglobin level, alanine aminotransferase (ALT), aspartate aminotransferase (AST), lipase, total bilirubin level, lymphocyte count, neutrophil count, and platelet count. Moreover, subgroup analysis was performed to classify hyperglycemia according to DAA experiences, non-cirrhosis and cirrhosis patients, intervention regimens, HCV GTs, and potential risk factors.

### 2.6. Quality assessment

Version 2 of the Cochrane risk-of-bias tool for randomized trials (RoB 2) was applied to determine the quality of each included study [27]. Two independent reviewers (VA and VB) evaluated the methodological quality of the selected studies, and a third reviewer (VV) resolved any discrepancies between the first two reviewers.

### 2.7. Certainty assessment

We assessed the certainty of the evidence for each article by using the Grading of Recommendations, Assessment, Development, and Evaluations (GRADE) framework, which classifies the quality of evidence as high, moderate, low, or very low. In the GRADE system, RCTs are considered to be high-quality evidence until downgraded based on five groups of limitations: risk of bias, imprecision, inconsistency (heterogeneity), indirectness, and reporting bias [28].

### 2.8. Statistical analyses

This research contained integrated data from the available articles. Findings were presented as risk ratios (RRs) with 95% confidence intervals (CIs) and incidence risk ratio (IRR). We also conducted sensitivity analyses to explore the impact of heterogeneity within our analysis. After removing studies that had a high risk of bias, we repeated our analyses by using fixed-effect models. All analyses were performed by using random-effects meta-analysis models with OpenMeta[Analyst] software (Center for Evidence-Based Medicine, Brown University, Rhode Island, USA). The R software (version 4.1; Camp Pontanezen, New Jersey, USA) was used to evaluate potential publication bias *via* a funnel plot.

## 3. Results

### 3.1. Literature selection and basic information

**Figure 1.**
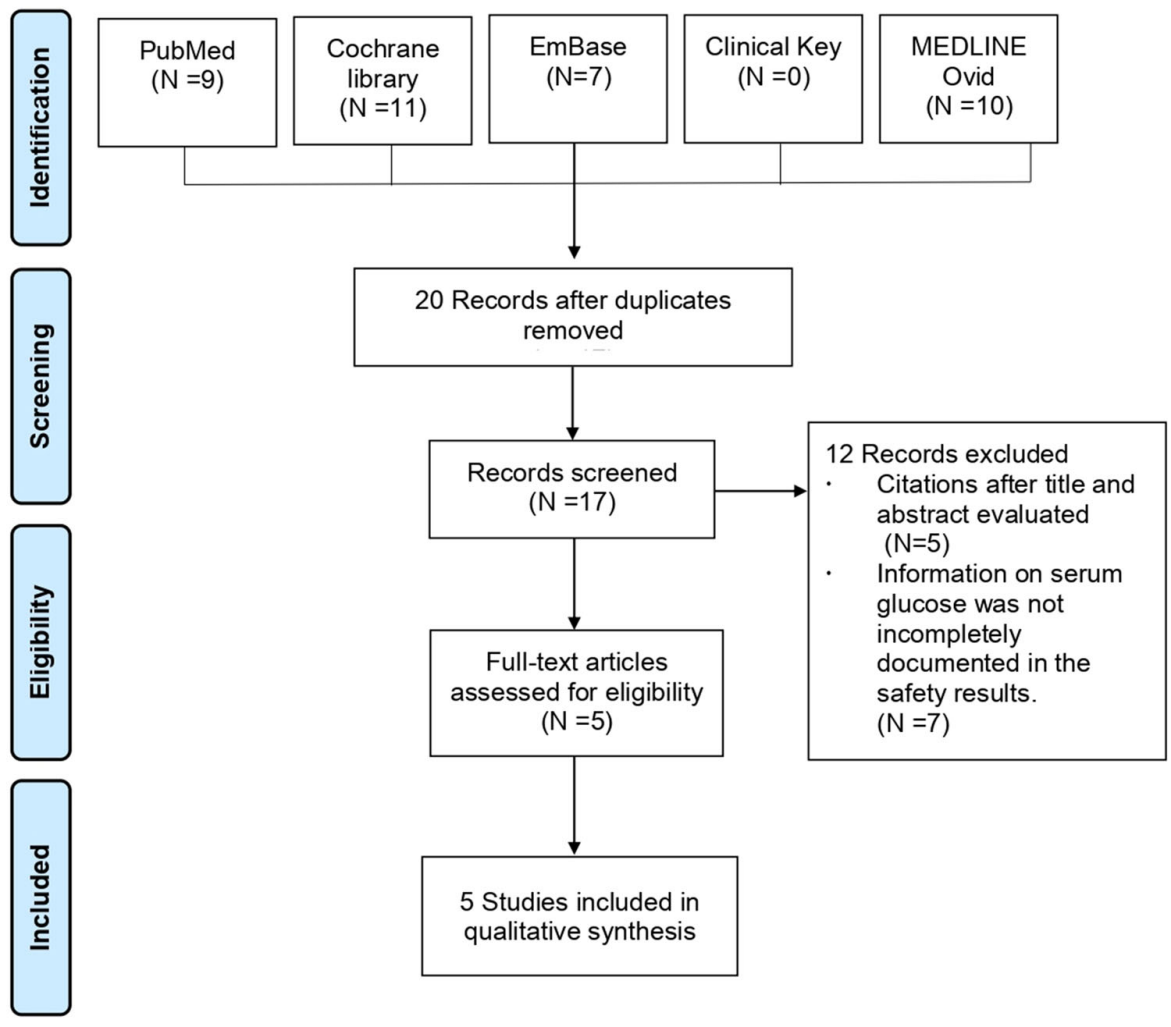
Flowchart of the identification of eligible trials.

The initial literature search identified a total of 37 articles, 32 of which were removed for being duplicates (*n*  =  20), having irrelevant titles and abstracts (*n*  =  5), and lacking grade 3 serum glucose outcomes (*n*  =  7). Data for the present systematic review were available from five studies with a total of 2315 HCV patients. The PRISMA flow diagram for the study selection process is presented in Figure 1.

The five studies [3,24,29–31] included in this review involved 2315 patients with or without compensating cirrhosis. All studies were open-label trials: phase II (*n*  =  2) [29,31] and phase III (*n*  =  3) [3,24,30]. HCV GT classifications in the included studies were as follows: HCV GTs 1–6 [3,24], HCV GT 1 [29,31], and HCV GT 1 or 3 [30]. In three different studies, the inclusion population had virologic failure who were treatment-naïve patients prior to DAA drug administration [3,24,30]. Most of the patients in the included studies were male and white and had cirrhosis. The details are presented in Table 1.

**Table 1.** Summary and baseline data of patients in included studies.

Author	Published years	Study design	Study name	HCV genotype, *n* (%)		Intervention	Week	Median age (range) years	Gender Male, *n* (%)	Race White, *n* (%)
Bourlière et al. [3]	2017	An open-label phase 3 trial study	POLARIS-1	GT1–6	GT1 150 (99)	Matched placebo	12	59 (29–80)	121 (80)	124 (82)
					GT1 150 (57)	SOF-VEL-VOX	12	58 (27–84)	200 (76)	211 (80)
			POLARIS-4	GT1–6	GT1 78 (43)	SOF-VEL-VOX	12	57 (24–85)	143 (79)	160 (88)
					GT1 66 (44)	SOF-VEL	12	57 (24–80)	114 (75)	131 (87)
Lawitz et al. [31]	2017	A phase 2, open-label study	–	GT1	GT1 (100)	SOF-VEL-VOX	12	54 (18–71)	16 (67)	17 (71)
					GT1 (100)	SOF-VEL-VOX + RBV	12	54 (22–75)	16 (64)	22 (88)
Jacobson et al. [24]	2017	In two phase 3, open-label trials	POLARIS-2	GT1–6	GT1 233(47)	SOF-VEL-VOX	8	53 (18–78)	255 (51)	391 (78)
					GT1 232(53)	SOF-VEL	12	55 (19–82)	237 (54)	365 (83)
			POLARIS-3		GT3 110 (100)	SOF-VEL-VOX	8	54 (25–75)	74 (67)	100 (91)
					GT3 109 (100)	SOF-VEL	12	55 (31–69)	100 (92)	97 (89)
Gane et al. [30]	2016	An open-label study	–	GT1, 3	GT1a 11 (73)	SOF-VEL-VOX	4	54 (40, 64)	9 (60)	12 (80)
					GT1a 11 (73)	SOF-VEL-VOX	6	50 (24, 65)	7 (47)	14 (93)
					GT1a 14 (93)	SOF-VEL-VOX	6	59 (51, 66)	11 (73)	14 (93)
					GT1a 23 (77)	SOF-VEL-VOX	6	55 (35, 73)	24 (80)	27 (90)
					GT1a 15 (88)	SOF-VEL-VOX	8	58 (48, 70)	14 (82)	16 (94)
					GT1a 24 (86)	SOF-VEL-VOX	8	57 (39, 66)	19 (68)	24 (86)
					GT3 18 (100)	SOF-VEL-VOX	6	52 (39, 64)	10 (56)	12 (67)
					GT3 19 (100)	SOF-VEL-VOX	8	55 (44, 66)	15 (79)	18 (95)
					GT3 4 (100)	SOF-VEL-VOX	8	56 (43, 62)	4 (100)	3 (75)
Lawitz et al. [29]	2016	An open-label, two-cohort, phase 2 study	–	GT1	GT1a 27 (79)	SOF-VEL-VOX	6	53	23 (68)	31 (91)
					GT1a 28 (78)	SOF-VEL-VOX	8	51	21 (58)	32 (89)
					GT1a 26 (79)	SOF-VEL-VOX	8	58	19 (58)	25 (76)
					GT1a 25 (81)	SOF-VEL-VOX + RBV	8	59	19 (61)	26 (84)
					GT1a 27 (87)	SOF-VEL-VOX	12	57	23 (74)	27 (87)
					GT1a 24 (75)	SOF-VEL-VOX	12	59	26 (81)	26 (81)

Author	Published years	Intervention	Week	Patients	Cirrhosis, no. (%)	Mean HCV RNA SD (log10 IU/ml)	Prior DAAs by class, *n* (%)					Relapse, *n* (%)	SVR12, % (95% CI)	Grade 3 Hyperglycemia^§^, n (%)
							NS5A + NS3 ± NS5B	NS5B ± NS3	NS5A ± NS5B	Treatment -naïve	Others			
Bourlière et al. [3]	2017	matched placebo	12	152	51 (34)	6.3 ± 0.6	62 (41)	1 (1)	80 (53)	–	–	–	–	7 (4.61)
		SOF-VEL-VOX	12	263	121 (46)	6.3 ± 0.7	83 (32)	0	161 (61)	–	–	6/261 (2)	96 (93.6–98.4)	5 (1.90)
		SOF-VEL-VOX	12	182	84 (46)	6.3 ± 0.6	0	46 (25)	0	–	–	1/182 (1)	98 (96–100)	4 (2.20)
		SOF-VEL	12	151	69 (46)	6.3 ± 0.7	0	38 (25)	0	–	–	14/150 (9)	90 (85–95)	3 (1.99)
Lawitz et al. [31]	2017	SOF-VEL-VOX	12	24	11 (46)	6.2 6 ± 0.42	6 (25)	14 (58)	4(16)	–	–	0	100 (86–100)	0 (0)
		SOF-VEL-VOX + RBV	12	25	14 (56)	6.3 6 ± 0.46	4 (16)	15 (60)	6 (24)	–	–	1 (4)	96 (80–100)	1 (4.00)
Jacobson et al. [24]	2017	SOF-VEL-VOX	8	501	90 (18)	6.1 (0.75)	501	–	–	383 (76)	–	21 (4)	95 (93–97)	7 (1.40)
		SOF-VEL	12	440	84 (19)	6.2 (0.66)	440	–	–	340 (77)	–	3 (1)	98 (97–99)	3 (0.68)
		SOF-VEL-VOX	8	110	110 (100)	6.0 (0.80)	110	–	–	75 (68)	–	2 (2)	96 (92–100)	8 (7.27)
		SOF-VEL	12	109	109 (100)	6.3 (0.63)	109	–	–	77 (71)	–	1 (1)	96 (92–100)	3 (2.75)
Gane et al. [30]	2016	SOF-VEL-VOX	4	15	0	6.3 (0.5)	–	–	–	15 (100)	–	11 (73)	27 (8–55)	0 (0)
		SOF-VEL-VOX	6	15	0	6.0 (0.7)	–	–	–	15 (100)	–	1 (7)	93 (68–100)	0 (0)
		SOF-VEL-VOX	6	15	15 (100)	6.0 (0.9)	–	–	–	15 (100)	–	2 (13)	87 (60–98)	1 (6.67)
		SOF-VEL-VOX	6	30	5 (17)	6.3 (0.5)	–	–	–	–	DAA	10 (30)	67 (47–83)	0 (0)
		SOF-VEL-VOX	8	17	17 (100)	6.3 (0.5)	–	–	–	–	PEG + RBV	0	100 (81–100)	0 (0)
		SOF-VEL-VOX	8	28	11 (39)	6.1 (0.6)	–	–	–	–	PI	3 (11)	89 (72–98)	1 (3.57)
		SOF-VEL-VOX	6	18	18 (100)	6.1 (0.7)	–	–	–	18 (100)	–	2 (11)	83 (59–96)	0 (0)
		SOF-VEL-VOX	8	19	19 (100)	6.3 (0.4)	–	–	–	–	PEG + RBV	0	100 (82–100)	3 (15.79)
		SOF-VEL-VOX	8	4	2 (50)	6.9 (0.2)	–	–	–	–	DAA	0	100 (40–100)	1 (25.00)
Lawitz et al. [29]	2016	SOF-VEL-VOX	6	34	0	6.2 (0.51)	–	–	–	34 (100)	–	10 (29)	71 (53–85)	0 (0)
		SOF-VEL-VOX	8	36	0	6.2 (0.55)	–	–	–	36 (100)	–	0	100 (90–100)	0 (0)
		SOF-VEL-VOX	8	33	33 (100)	6.0 (0.59)	–	–	–	33 (100)	–	2 (6)	94 (80–99)	1 (3.03)
		SOF-VEL-VOX + RBV	8	31	31 (100)	6.3 (0.47)	–	–	–	31 (100)	–	6 (19)	81 (63–93)	1 (3.23)
		SOF-VEL-VOX	12	31	0	6.4 (0.77)	17(55)	14 (45)	–	–	–	0	100 (89–100)	0 (0)
		SOF-VEL-VOX	12	32	32 (100)	6.0 (0.61)	12 (38)	20 (62)	–	–	–	0	100 (94–100)	0 (0)

HCV: hepatitis C virus; GT: genotype; SOF: Sofosbuvir; VEL: velpatasvir;VOX: voxilaprevir; RBV: ribavirin; SD: standard deviation; NS5A: Nonstructural protein 5 A inhibitors; NS3: NS3 protease inhibitors; NS5B: Non-structural protein 5B inhibitors; DAAs: direct acting antiviral agents; SVR12: sustained virologic response after treatment 12 weeks; 95% CI, confidence interval; § Grade 3 Hyperglycemia, serum glucose >250 mg/dl.

Other grade 3 laboratory abnormalities in the levels of Hb, lymphocytes, neutrophils, platelets, ALT, AST, lipase, and bilirubin are shown in Table 2.

**Table 2.** Others grade 3 laboratory abnormality.

Author	Published years	Intervention	Week	Patients	Hemoglobin level <10 g/dl *n* (%)	Lymphocyte count <500/mm^3^ *n* (%)	Neutrophil count <750/mm^3^ *n* (%)	Platelet count <50,000/mm^3^ *n* (%)	ALT >5 × ULN *n* (%)	AST >5 × ULN *n* (%)	Lipase level >3 × ULN *n* (%)	Total bilirubin level >2.5 × ULN (Hyperbilirubinemia) *n* (%)
Bourlière et al. [3]	2017	matched placebo	12	152	1 (0.66)	2 (1.32)	0 (0)	0 (0)	3 (1.97)	7 (4.61)	4 (2.63)	0 (0)
		SOF-VEL-VOX	12	263	2 (0.76)	0 (0)	1 (0.38)	2 (0.76)	0 (0)	2 (0.76)	6 (2.28)	1 (0.38)
		SOF-VEL-VOX	12	182	2 (1.10)	1 (0.55)	0 (0)	3 (1.65)	1 (0.55)	0 (0)	3 (1.65)	0 (0)
		SOF-VEL	12	151	1 (0.66)	1 (0.66)	2 (1.32)	2 (1.32)	0 (0)	0 (0)	1 (0.66)	0 (0)
Lawitz et al. [31]	2017	SOF-VEL-VOX	12	24	0 (0)	0 (0)	–	1 (4.17)	0 (0)	–	0 (0)	–
		SOF-VEL-VOX + RBV	12	25	6 (24.00)	1 (4.00)	–	1 (4.00)	1 (4.00)	–	1 (4.00)	–
Jacobson et al. [24]	2017	SOF-VEL-VOX	8	501	6 (1.20)	1 (0.20)	2 (0.40)	3 (0.60)	0 (0)	1 (0.20)	5 (1.00)	0 (0)
		SOF-VEL	12	440	3 (0.68)	3 (0.68)	2 (0.45)	2 (0.45)	1 (0.23)	0 (0)	3 (0.68)	1 (0.23)
		SOF-VEL-VOX	8	110	3 (2.73)	3 (2.73)	0 (0)	1 (0.91)	0 (0)	1 (0.91)	2 (1.82)	0 (0)
		SOF-VEL	12	109	0 (0)	1 (0.92)	1 (0.92)	1 (0.92)	0 (0)	1 (0.92)	1 (0.92)	0 (0)
Gane et al. [30]	2016	SOF-VEL-VOX	4	15	–	–	0 (0)	0 (0)	–	0 (0)	0 (0)	–
		SOF-VEL-VOX	6	15	–	–	0 (0)	0 (0)	–	0 (0)	0 (0)	–
		SOF-VEL-VOX	6	15	–	–	1 (6.67)	0 (0)	–	0 (0)	1 (6.67)	–
		SOF-VEL-VOX	6	30	–	–	0 (0)	0 (0)	–	1 (3.33)	3 (10.00)	–
		SOF-VEL-VOX	8	17	–	–	0 (0)	1 (5.88)	–	1 (5.88)	0 (0)	–
		SOF-VEL-VOX	8	28	–	–	0 (0)	0 (0)	–	0 (0)	1 (3.57)	–
		SOF-VEL-VOX	6	18	–	–	0 (0)	1 (5.56)	–	0 (0)	1 (5.56)	–
		SOF-VEL-VOX	8	19	–	–	1 (5.26)	0 (0)	–	0 (0)	1 (5.26)	–
		SOF-VEL-VOX	8	4	–	–	0 (0)	0 (0)	–	0 (0)	0 (0)	–
Lawitz et al. [29]	2016	SOF-VEL-VOX	6	34	0 (0)	–	–	0 (0)	0 (0)	–	–	0 (0)
		SOF-VEL-VOX	8	36	0 (0)	–	–	0 (0)	0 (0)	–	–	0 (0)
		SOF-VEL-VOX	8	33	0 (0)	–	–	1 (3.03)	0 (0)	–	–	–
		SOF-VEL-VOX + RBV	8	31	4 (12.90)	–	–	0 (0)	1 (3.23)	–	–	3 (9.68)
		SOF-VEL-VOX	12	31	0 (0)	–	–	0 (0)	0 (0)	–	–	0 (0)
		SOF-VEL-VOX	12	32	0 (0)	–	–	2 (6.25)	0 (0)	–	–	1 (3.13)

SOF: Sofosbuvir; VEL: velpatasvir; VOX: voxilaprevir; RBV: ribavirin; ALT: alanine aminotransferase; AST: aspartate aminotransferase; ULN: upper limit of normal.

### 3.2. Grade 3 hyperglycemia incidence

**Figure 2.**
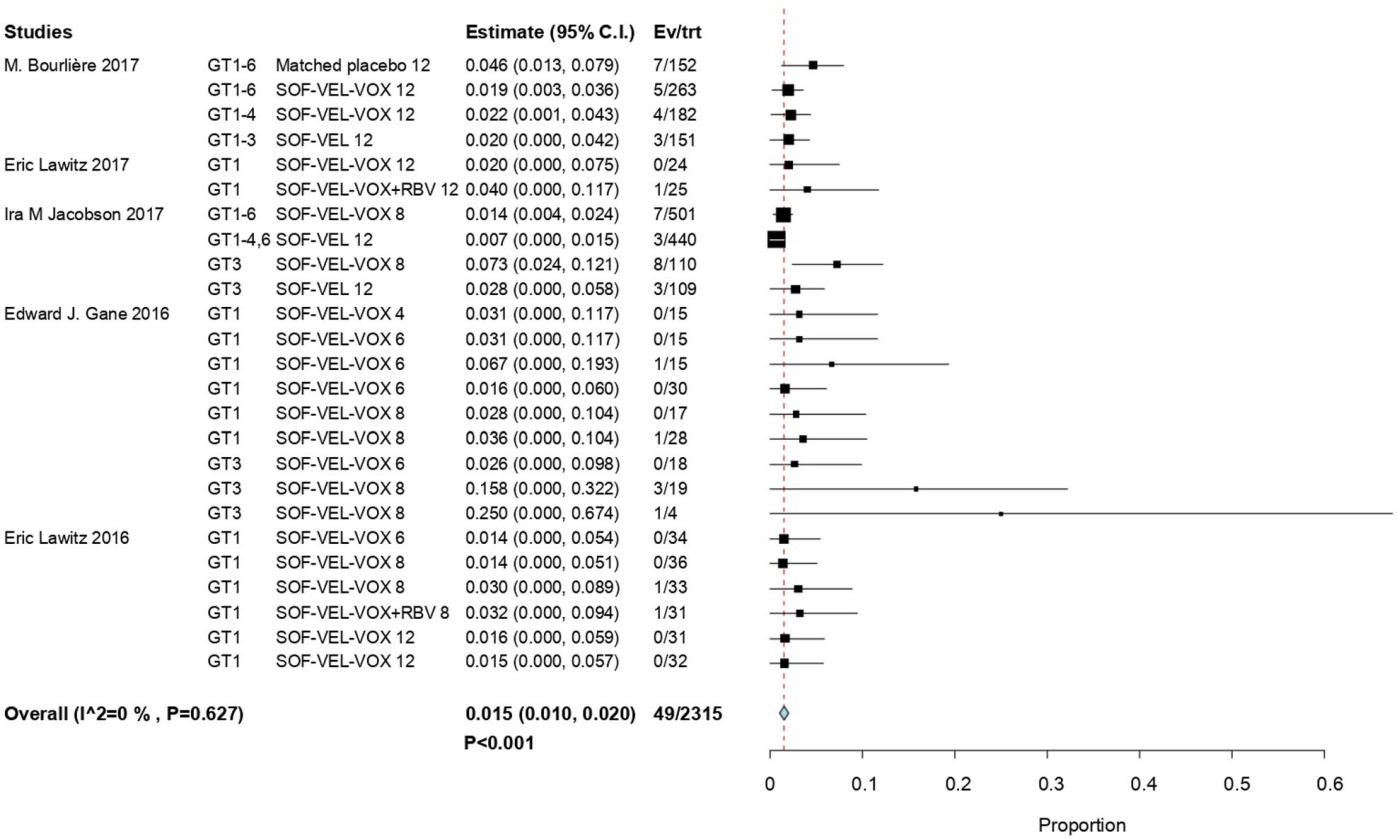
Forest plots describing the incidence of grade 3 hyperglycemia following SOF/VEL/VOX therapy.

Grade 3 level hyperglycemia was observed in 49 of 2315 patients, with an overall RR of 0.015 (95% CI, 0.010–0.020; *p*  <  .001; *I*^2^  =  0%). HCV GT 3 infections had the highest RR at 0.25 (95% CI, 0.00–0.674); it had higher frequency of occurrence than the other HCV GT infections. This was attributed to differences in study populations and the association of glucose dysregulation with liver disease [24]. Another study on serum glucose reported the case with asymptomatic known diabetes [30]. The findings of this study revealed that there was no significant effect related to combined ribavirin (RBV) [29,31]. The incidence of hyperglycemia was not a significant influence on efficacy outcomes for SOF/VEL/VOX (Figure 2).

### 3.3. Sensitivity analysis

**Figure 3.**
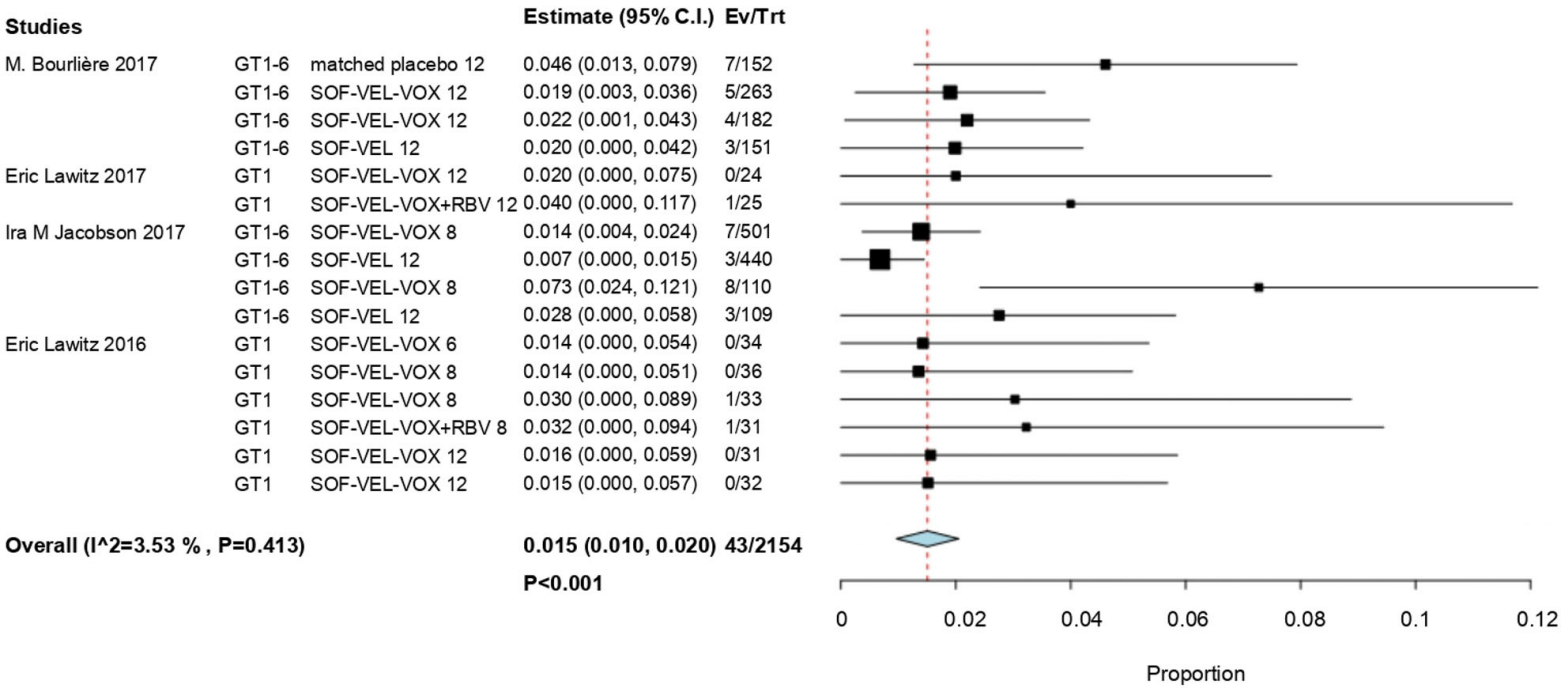
The forest plots of sensitivity analysis.

A sensitivity analysis was performed by excluding a study [30] due to trial duration (< 12 weeks of standard therapy) and risk of bias (a study with a high risk of bias across overall domains). Next, we repeated our analyses by using fixed-effects models. The results showed that the RR value was not affected and remained at 0.015 (95% CI, 0.010–0.020; *p*  <  .001) (Figure 3).

### 3.4. Meta-regression and subgroup analysis in grade 3 hyperglycemia

Meta-regression was used to investigate potential factors affecting the heterogeneity of intervention regimens, examine different GTs, and assess study effect sizes (Supplementary Table S2). After adjusting for HCV GT 1 and standard therapy with SOF/VEL/VOX for 12 weeks, we found that the incidence of hyperglycemia was not affected by different GTs and treatment regimens, with no statistically significant difference (*p*  >  .05).

**Figure 4.**
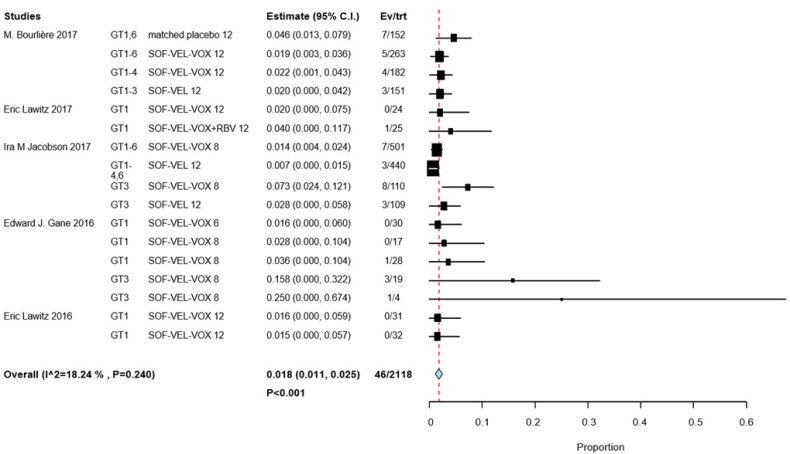
Forest plots for previously treated patients in subgroup analysis.

In total, 46 of the 2118 patients with prior treatment experience were treated with SOF/VEL/VOX (RR, 0.018; 95% CI, 0.011–0.025; *p*  <  .001) (Figure 4).

**Figure 5.**
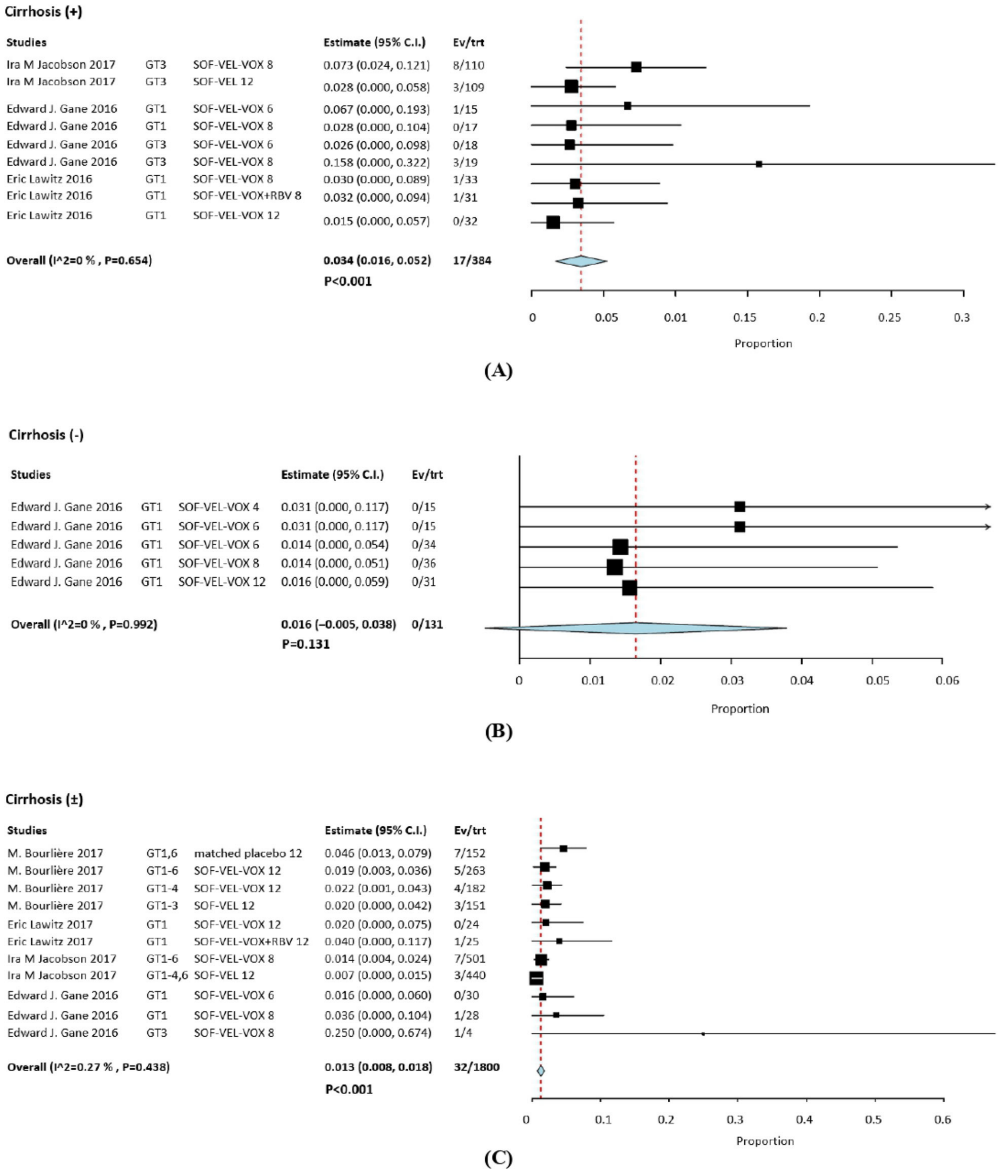
Subgroup analysis in hyperglycemia among with and without cirrhosis group. (A) cirrhosis, (B) without cirrhosis, (C) with and without cirrhosis.

Analysis of the incidence of patients treated with SOF/VEL/VOX showed that 17/384 patients with cirrhosis (RR: 0.034; 95% CI, 0.016–0.012; *p*  =  .052; *I*^2^ = 0%, *p*  < .001), whereas 0/131 patients without cirrhosis (RR, 0.16; 95% CI, −0.005 to 0.012; *p*  =  .038; *I*^2^ = 0%, *p*  =  .131), and 32/1800 patients with and without cirrhosis (RR, 0.013; 95% CI, 0.008–0.012; *I*^2^ = 0.27%, *p* < .001) (Figure 5). With cirrhosis and with/without cirrhosis groups compared with non-cirrhosis IRR were 12.000 (95% CI, 0.727–198.160) and 4.764 (95% CI, 0.293–77.366), respectively (Supplementary Table S3). The IRR for cirrhosis versus with/without cirrhosis was 2.490 (95% CI, 1.397–4.438).

**Figure 6.**
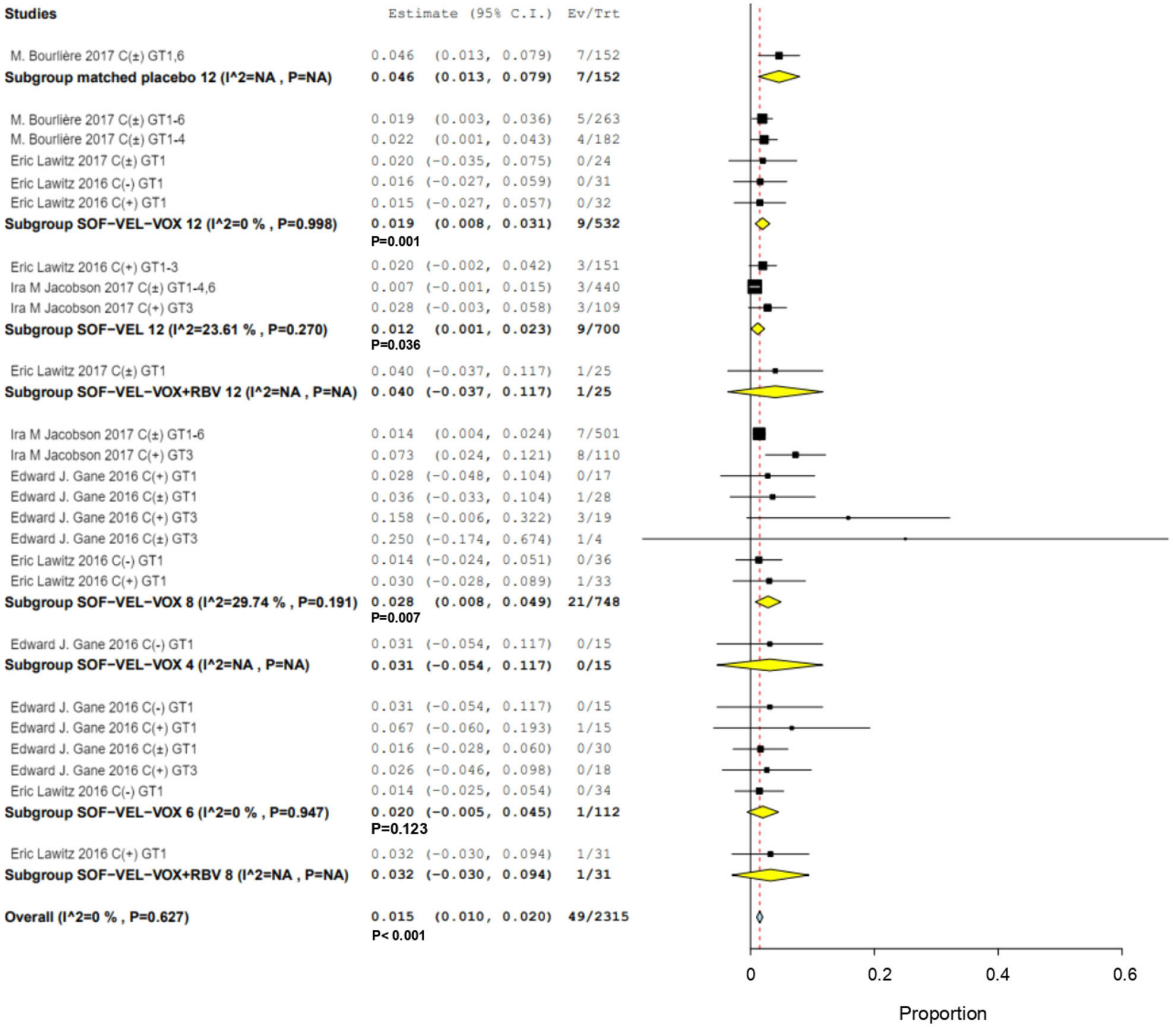
Subgroup analysis by grade 3 event of varying interventions.

Subgroup analyses by interventions and HCV GTs revealed that 9 of 700 patients in two studies received SOF/VEL for 12 weeks (RR, 0.012; 95% CI, 0.001–0.012; *p*  =  .036; *I*^2^ = 23.61%, *p*  =  .270); 1 of 122 patients with HCV GTs 1 and 3 in 2 studies received SOF/VEL/VOX for 6 weeks (RR, 0.002; 95% CI, −0.005 to 0.045; *p*  =  .123); 9 of 532 patients in three studies received SOF/VEL/VOX for 12 weeks (RR, 0.019; 95% CI, 0.008–0.031; *p*  =  .001; *I*^2^ = 0%, *p*  =  .998); and 21 of 748 patients in three studies received SOF/VEL/VOX for 8 weeks (RR, 0.028; 95% CI, 0.008–0.049; *p*  =  .007; *I*^2^ = 29.74%, *p*  =  .191). No significant differences were observed with 8-week treatments compared to 12-week treatments, the IRR was 1.66 (95% CI: 0.77–3.60) (Figure 6).

**Figure 7.**
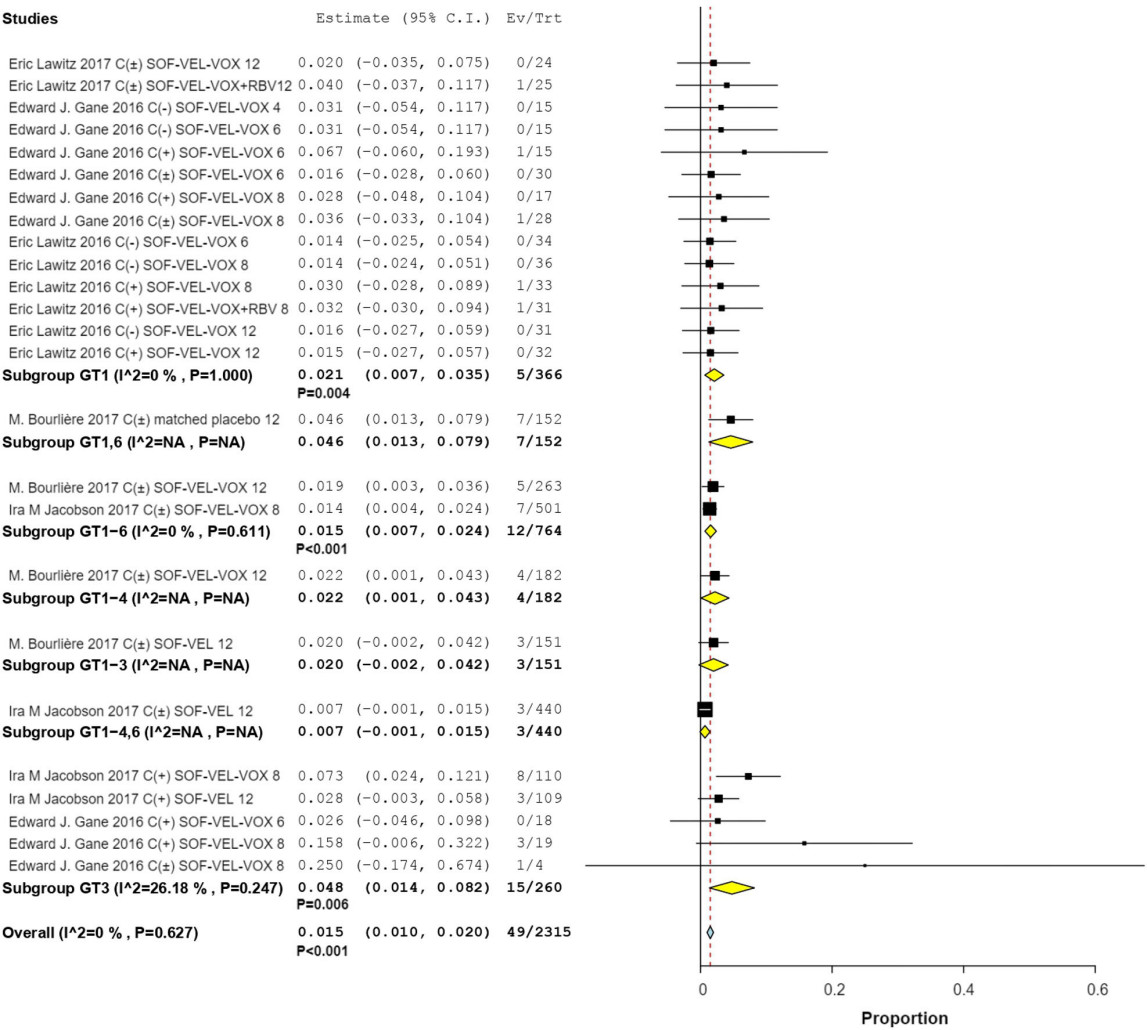
Subgroup analysis by different hepatitis C genotypes.

Among the patients whose HCV GTs were available for analysis, 5/366 had HCV GT 1 infection (RR, 0.021; 95% CI, 0.007–0.035; *p*  = .004), 12/764 had HCV GTs 1–6 infections (RR, 0.015; 95% CI, 0.007–0.024; *p* <  .001), and 15/260 had HCV GT 3 infections (RR, 0.048; 95% CI, 0.014–0.082; *p*  =  .06; *I*^2^ = 26.18%, *p* =  .247). The incidence of hyperglycemia was higher in patients with HCV GT 3 than in those with HCV GT 1, the IRR was 4.13 (95% CI: 1.52–11.22) (Figure 7).

Potential high-risk factors were identified from the analysis of the above subgroups: HCV GT3 and cirrhosis. Hence, we further assessed the HCV GT3 with cirrhosis subgroup in the available articles. In two studies, three regimens, involving HCV GT3/cirrhotic participants, whose hyperglycemia occurred in 11/147 patients and RR: 0.064 (95% CI, 0.017–0.111; *p*  = .007). With cirrhosis and HCV GT3 compared to HCV cirrhotic/GT3 group IRR were 0.592 (95% CI, 0.284–1.233) and 0.771 (95% CI, 0.364–1.634), respectively. No statistically significant differences were observed, although the HCV cirrhotic/GT3 has a higher RR value in Supplementary Figure S1 and Table S3.

### 3.5. Other SOF/VEL/VOX-induced grade 3 laboratory abnormalities

In August 2019, the U.S. Food and Drug Administration published a safety announcement about the rare occurrence of serious liver injury in HCV patients with moderate to severe liver impairment following treatment with SOF/VEL/VOX. By 8 January 2019, there were three cases of liver decompensation in the FDA Adverse Event Reporting System database and in the medical literature after SOF/VEL/VOX therapy for HCV. These events generally occurred within the first 4 weeks of treatment. Most patients had improved symptoms or liver function after discontinuing the treatment [32]. In the pooled analysis, grade 3 or 4 laboratory abnormalities were infrequent in patients receiving SOF/VEL/VOX. There were rare cases of grade 3 or 4 elevations in ALT, AST, and total bilirubin [4].

In the POLARIS-1 and POLARIS-4 studies, asymptomatic lipase levels of higher than three times the ULN were observed in 2% of the patients treated with SOF/VEL/VOX [3]. Moreover, Hb abnormalities were mainly found in patients receiving RBV 24.00% and 12.90% [29,31], and other grade 3 treatment-related abnormalities in laboratory parameters are integrated in Table 2. In this study, other grade 3 laboratory abnormalities have a lower incidence compared to hyperglycemia (Supplementary Table S3), while it involves a potential selection bias.

### 3.6. Quality assessment and risk of bias

**Figure 8.**
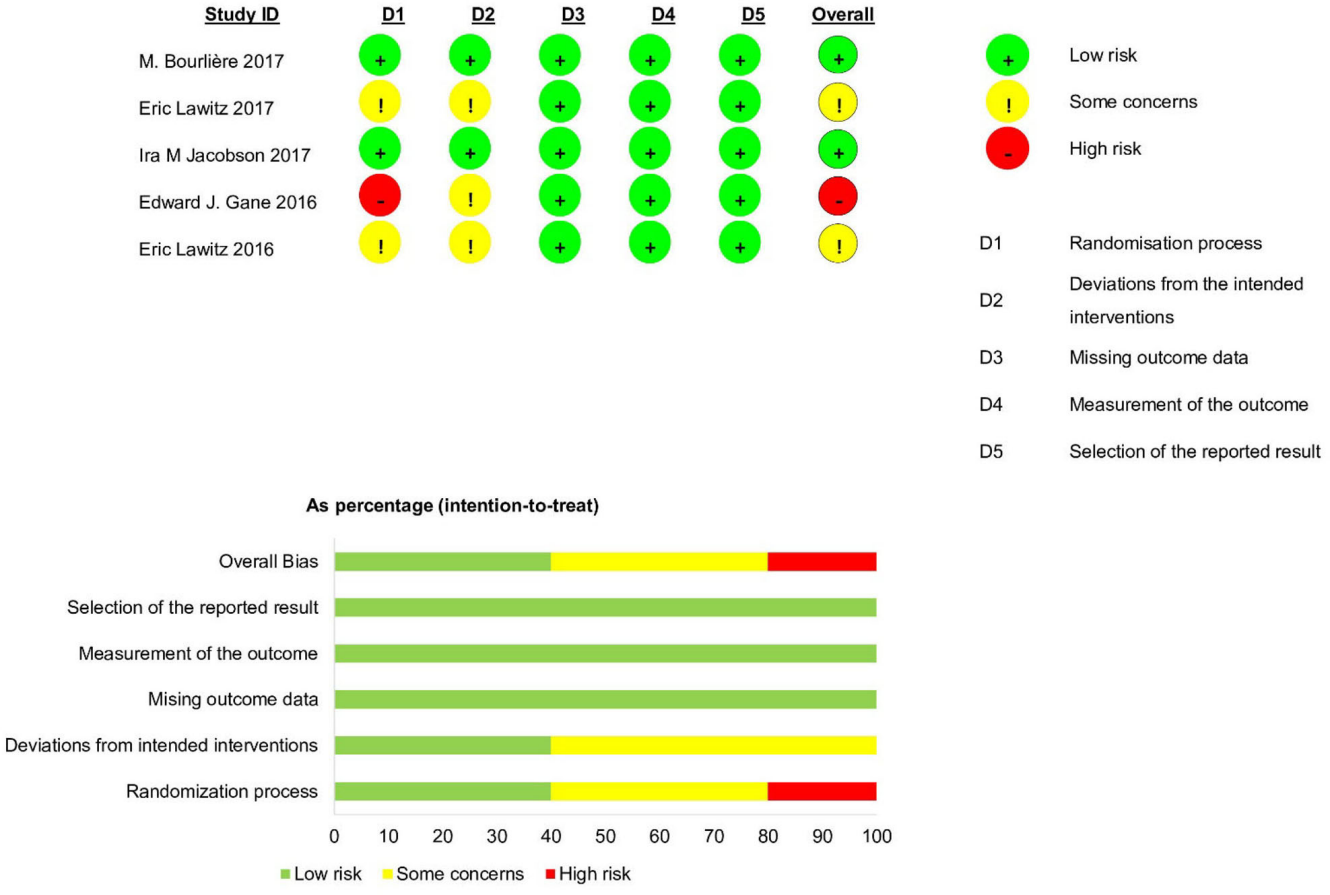
Risk of bias for available studies.

Using the RoB 2.0 tools, one study [30] was evaluated as ‘high risk of bias’ due to failing to mention random allocation and two others [29,31] as ‘some concerns’ across the overall domain because most studies were open-label trials focused on HCV infections. However, the deviations resulting from open-label trials were unlikely to have affected the efficacy outcome (Figure 8).

### 3.7. Reporting bias and certainty of evidence

Since the number of included articles was less than 10, the funnel plot was inappropriate to assess the publication deviation in Supplementary Figure S2. The certainty of evidence (GRADE) was very low for the outcome. We predominantly downgraded the certainty as all studies were open-label trials and the event rate was extremely small. Hence, RCTs were rated down due to limitations related to the risk of bias, indirectness, and imprecision although they began as high-quality evidence (Supplementary Table S4).

## 4. Discussion

In the five enrolled RCTs, progress to grade 3 hyperglycemia was rare (overall RR, 0.015; 95% CI, 0.010–0.020), although this finding was contrary to the majority of previous studies for hypoglycemia. In subgroups analysis, with cirrhosis compared to without cirrhosis and no mentioned cirrhosis status groups IRR were 12.000 (95% CI, 0.727–198.160) and 2.490 (95% CI, 1.397–4.438), respectively. HCV GT3 infections compared with HCV GT1 infections IRR was 4.128 (95% CI, 1.519–11.218). And, no significant differences were observed in the DAA experiences and intervention regimens subgroup analysis. Two of five eligibility articles have explained the hyperglycemia results, which included asymptomatic known diabetes. Other, the participants were not mentioned as diabetic. According to the drug safety announcement was recommended that glucose levels be closely monitored within the first 3 months for diabetics and that their diabetes medications or doses be adjusted as necessary.

We assessed factors likely to affect serum glucose. Further searches were conducted through the Cochrane Central Register of Controlled Trials (CENTRAL) and Clinical Trial.gov for information about adverse effects caused by pharmacological mechanisms or dose-dependent reactions, with the last search conducted on December 30, 2020. No association was found between high blood sugar and the medical components of SOF/VEL/VOX. In clinical studies comparing different interventions with SOF/VEL, only three studies that illustrated safety events reported hyperglycemia [33–35]. Firstly, grade 3 glucose abnormalities were detected in two patients (1.80%) in the ASTRAL-1 study [33]. Secondly, these abnormalities were observed in Japanese patients with HCV GTs 1–3 and decompensated cirrhosis who underwent 12-week treatment with SOF/VEL (9.80%) and 12-week treatment with SOF/VEL + RBV (17.65%) [34]. This study did not provide any explanation regarding hyperglycemia and its associated factors. A third study reported a history of diabetes in eight Japanese patients with grade 3 hyperglycemia in whom DAA therapy for HCV GT 1 or 2 infections had already failed [35]. Additionally, attempts to search for clinical trials correlating VOX with blood glucose abnormalities were made, but no data were acquired. Consequently, we considered the strong possibility that the lactose-containing excipients of SOF/VEL/VOX could serve as an influencing factor, leading to serum glucose abnormalities [8]. Evidence from an animal trial exploring the effect of lactose on plasma glucagon-like peptide (GLP)-1 and GLP-2 concentrations and gastrointestinal tract development in Holstein bull calves suggested that lactose inclusion in calf starters contributes to the maintenance of high plasma GLP-1 and GLP-2 concentrations. Moreover, the amount of lactose added was proportional to the rate of increase in plasma GLP-1 concentrations, and the plasma GLP-1 concentration was positively correlated with insulin concentration (*r* = 0.793) [36]. Admittedly, in addition to viral infection and disease control, other factors should be considered while interpreting glucose homeostasis parameters in these patients.

The pathogenetic link is complicated between IR and chronic hepatitis C. IR encourages lipid accumulation, inflammation, and fibrosis precipitation in the liver with illness progression. On the other hand, HCV may own induce into IR, with T2DM advancement and lipid metabolism insanity [37]. Our study concludes that the following reasons might rationally explain that in genotype 3 and cirrhosis might be linked around hyperglycemia occur. Chronic HCV infection cirrhotic patients have a higher risk for developing T2DM. A study showed that the prevalence of T2DM in a group of chronic hepatitis C patients with cirrhosis is higher than in both groups, not hepatic disease and liver cirrhosis owing to other causes. In HCV patients, new onset diabetes predicts decompensation of liver cirrhosis (RR: 2.01; 95% CI: 1.07–3.79; *p* < .001) [38]. On the other hand, at the Initialing of the DAA era, GT3 emerged as a ‘difficult-to-treat’ GT. HCV GT3 was defined through a steatosis has a higher rate, advance progression of liver fibrosis, hepatic cirrhosis risk rising, and comparing with other HCV GTs infection has a lower efficacy of antiviral therapy. Moreover, the advancement for GT3 infection was not as pronounced as for other GTs, especially in prior-experience treated patients and liver cirrhosis [39]. In HCV infection with other genotypes, hepatic steatosis and IR are thought to be primarily owing to changes in host metabolism. Instead, hepatic steatosis is most usual in HCV GT3, perhaps because of the direct effects of GT3 viral proteins, resume after achieving the SVR and reoccurs in relapses. Three dissimilar non-mutually exclusion mechanisms leading to HCV GT3 modulates lipid metabolism and signaling: *via* impeded lipoprotein secretion or fatty acid degradation (β-oxidation) and *via* increased lipogenesis. Additionally, Molecular studies illustrated the capacity of HCV GT3 to encourage IR and T2DM. HCV can induce IR through directly disturb intra-cellular insulin signalling or the chronic inflammatory response induces it indirectly. All these alterations can block the transactivation of glucose transporters-4 in the cells, inhibiting glucose uptake lead to a hyperinsulinism status [37].

According to Health Canada’s announcement about the risk of hypoglycemia in patients with HCV/DM infection through DAA treatment [23]. This is a reasonable explanation due to HCV elimination for an enhanced hypoglycemic effect. Longer-term HCV infections have been known to increase the risk for diabetes. Successful eradication of HCV may help improve glycemic control in patients with diabetes [7], gradually decrease the need for insulin, and even achieve a reduced incidence of T2DM [40]. A study showed that these effects were greater in patients with mild liver disease (Child-Pugh score A) and had no relationship with age, gender, or body mass index [7]. Similar findings were observed in another study, where SOF/VEL/VOX reduced daily insulin doses and improved HbA1C changes [41]. Therefore, Health Canada advice for healthcare professionals, it is vigilant for blood glucose alteration during DAA therapy, especially within the first 3 months when rapid reduction in viral load, and modify diabetic prescription when necessity [23].

Regarding other hepatitis complications, grade 3 hyperglycemia occurred in two diabetic patients (2.6%) with HIV/HCV coinfection [42]. Patients with HIV/HCV coinfection are at elevated risk for metabolic syndrome and progression to T2DM and cardiovascular disease. HCV elimination by DAA therapy may improve metabolic comorbidities [43]. Nevertheless, a large cohort study of patients with HIV/HCV coinfection indicated that there was no difference in the rates of T2DM between well-defined HCV strata, including chronic and cured HCV [44]. In a hepatitis B population, treatment with interferon (IFN)-α for 48 weeks significantly redcued the mean homeostasis model assessment (HOMA)-IR score, followed by SVR achievement rather than virologic failure [45].

After a long-term follow-up, Ciancio et al. found significant reductions in fasting glucose and HbA1C values after 44.5 months [46]. However, two studies have reported a significant glycometabolic improvement in SVR [10], and they did not confirm the long-term improvement [47,48]. Patients with cirrhotic HCV/diabetes benefit only in the short term [49].

Considering the other DAA regimens, the effect of eradication of HCV infection by SOF and daclatasvir, a total of 37.6% of patients with diabetes showed a significant reduction of mean HbA1C [50]. Similar conditions were observed in treatment-naïve Egyptian patients with T2DM without cirrhosis [51] as well as in HCV GT 3 patients [52]. Likewise, treatment with SOF and ledipasvir was found to be accompanied by immediate amelioration in HbA1C [53]. In case of diabetic patients undergoing IFN-free and RBV-free antiviral treatment for HCV, HbA1C reductions were greater in the patients who achieved SVR than in those with persistent treatment failure [53]. A previous study found that DAA treatment led to a significant reduction in the post-Oral Glucose Tolerance Test plasma glucose concentration [19]. Additionally, some studies have revealed significant reductions in IR [11,17,18] and insulin plasma concentrations but an increase in insulin sensitivity at the end of treatment—despite a significant decline in insulin secretion [19]. Interestingly, HOMA-IR reductions occurred in diabetic patients who achieved SVR [17,54], implying that the benefits of viral eradication are not limited to diabetes and can be applied to the entire spectrum of glucose metabolism [19]. A review article reported that SVR achievement was associated with reduced IR at follow-up (OR, 0.42) and had a significant protective effect on the incidence of diabetes (OR, 0.34) [11].

Glycemic control benefits have also been observed with IFN-based therapies [16,55,56]. A meta-analysis suggested that there is a significant association between the effects of IFN-α treatment and SVR, which helps lower the risk of developing glucose abnormalities [57]. A trial reported persistent modest improvement in lipid profiles and IR with pegylated (PEG)-IFN-α and RBV in patients with HCV/HIV coinfection [58]. Another study by Serfaty et al. showed that after 12 weeks of treating treatment-naïve patients with telaprevir plus peg-IFN-α/RBV, the HOMA-IR score was significantly lower in patients with SVR than in those without SVR [59]. A study demonstrated that glycometabolism improved after viral eradication, which in turn decreased the fasting plasma glucose level in HCV patients receiving PEG-IFN-α and RBV. Yuan et al. demonstrated that glycometabolism improved after viral eradication, which in turn decreased the fasting plasma glucose level [14]. It has been proved that the eradication of HCV is associated not only with IR improvement after IFN-based therapy but also with a significant reduction in T2DM and its complications [18]. By contrast, a study in Brazil displayed no statistically significant difference between HOMA-IR scores in patients before and 12 months after treatment for HCV [60].

To state succinctly, an improvement in the glycometabolic control at the end of hepatitis C treatment or in the instant post-therapy months in diabetic patients, or only in some subgroups, has been reported by a wide majority of studies so far. Whether this advantageous effect is maintained over the long term is still a matter of debate. Hence, cautious longitudinal monitoring of these patients is needed before claiming any consolidated efficacy [10].

## 5. Strengths and weaknesses of this study

We have integrated high-quality RCT articles to examine the incidence of grade 3 hyperglycemia and to assess differences between adverse events and subgroups. Considering lactose-containing excipients, HCV genotypes, and cirrhosis are risk factors for progression to grade 3 hyperglycemia. These outcome was contrary to the majority of the literature of blood glucose change. Our study had some limitations. The effect size was restricted—regardless of pre-market clinical trials or real-world data—because the latest approved DAA regimens are mostly prescribed in developed countries. SOF/VEL/VOX was indicated for cases with previous DAAs failure; thus, the number of participants have been limited.

## 6. Conclusion

SOF/VEL/VOX for 12 weeks is a first rescue option for retreatment of HCV patients previously failing DAA therapy, regardless of the genotype, cirrhosis, and compensated patients. Patients with cirrhosis and HCV GT 3 infection have a high risk of grade 3 hyperglycemia although the incidence was found to be unusual in diabetic patients with HCV, it is recommended that glucose levels be closely monitored during the first 3 months of treatment with SOF/VEL/VOX and that diabetes medication be modified when necessary.

## Supplementary Material

Supplemental Material

## Data Availability

No data is available for this study.
