# Supplementary material for: The impact of sofosbuvir/velpatasvir/voxilaprevir treatment on serum hyperglycemia in hepatitis C virus infections: a systematic review and meta-analysis
Source: Ann Med. 2023 Jan 19;55(1):463–79. doi: 10.1080/07853890.2023.2168745 (PMC9858431; doi:10.1080/07853890.2023.2168745)
Supplement: Supplemental Material [file IANN_A_2168745_SM2504.docx]

**Supplement**

**Supplementary Figure S1** Forest plots for HCV genotype 3 with cirrhosis subgroup

**Supplementary Figure S2** Funnel plot for event rate

**Supplementary Table S1** Search strategy for each database

**Supplementary Table S2** Meta-regression

**Supplementary Table S3** Comparing risk ratio

**Supplementary Table S4** Certainty of evidence

This supplementary material has been provided by the authors to give readers

additional information about their work.


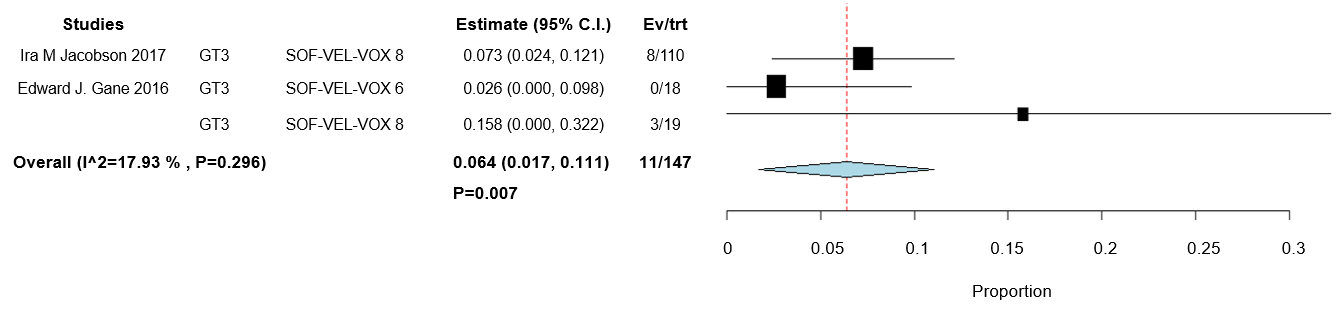


1. Forest plots for HCV genotype 3 with cirrhosis subgroup


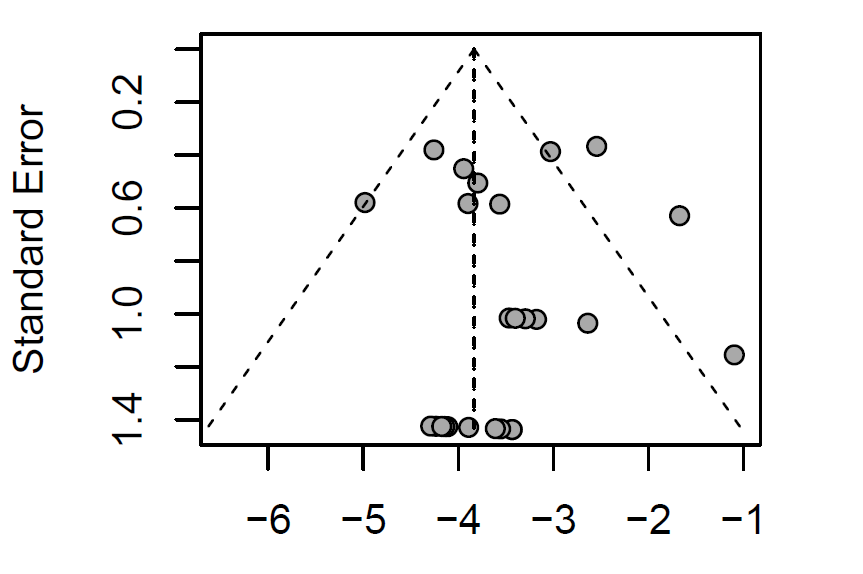


1. Funnel plot for event rate
2. Search strategy for each database

| Database | Search Strategy |
| --- | --- |
| Pubmed | ((hepatitis c[MeSH Terms]) OR (chronic hepatitis c[MeSH Terms])) AND ((((vosevi) OR ((sofosbuvir) AND (velpatasvir) AND (voxilaprevir))) OR (SOF/VEL/VOX)) AND (GS‐9857 OR voxilaprevir OR VOX)) |
| Embase | \|  \| All fields \| (hepatitis c ) AND (chronic hepatitis C) \| \| --- \| --- \| --- \| \| AND \| All fields \| (vosevi) OR ((sofosbuvir) AND (velpatasvir) AND (voxilaprevir)) \| \| AND \| All fields \| (voxilaprevir) OR (GS‐9857) OR (VOX) \|   **chronic** AND **hepatitis** AND **c** AND (**vosevi** OR (**sofosbuvir** AND **velpatasvir** AND **voxilaprevir**)) AND (**voxilaprevir** OR **GS‐9857** OR **VOX**) AND [randomized controlled trial]/lim |
| Cochrane | #1 AND #2 AND #3  #3 (hepatitis c ) AND (chronic hepatitis C)  #2 (vosevi) OR ((sofosbuvir) AND (velpatasvir) AND (voxilaprevir))  #1 (voxilaprevir) OR (GS‐9857) OR (VOX) |
| Clinical key | (hepatitis C OR chronic hepatitis C) AND (voxilaprevir OR VOX)  (SOF/VEL) AND (voxilaprevir OR VOX) AND (hepatitis C ) |
| MEDLINE-Ovid | (hepatitis c or chronic hepatitis C) AND (voxilaprevir or VOX) |

1. Meta-regression

| Random and fixed effects | |  |  |  |  |
| --- | --- | --- | --- | --- | --- |
| Metric: Proportion |  |  |  |  |  |
| Covariate | Level | Studies number | Coefficient (95% CI) | SE | P-value |
| Cirrhosis | Intercept |  | 0.014 (0.008, 0.02) | 0.003 | < 0.001 |
|  | Cirrhosis(±) | 11 | reference |  |  |
|  | Cirrhosis(-) | 5 | 0.002 (-0.02-0.025) | 0.011 | 0.835 |
|  | Cirrhosis(+) | 9 | 0.02 (0.001-0.039) | 0.01 | 0.037 |
|  |  |  |  |  |  |
| Genotype | Intercept |  | 0.015 (0.007-0.024) | 0.004 | < 0.001 |
|  | GT1-6 | 2 | reference | reference | reference |
|  | GT1 | 14 | 0.006 (-0.011- 0.023) | 0.009 | 0.515 |
|  | GT1-3 | 1 | 0.004 (-0.019- 0.028) | 0.012 | 0.713 |
|  | GT1-4 | 1 | 0.007 (-0.016- 0.03) | 0.012 | 0.574 |
|  | GT1-4,6 | 1 | -0.009 (-0.02- 0.003) | 0.006 | 0.149 |
|  | GT1,6 | 1 | 0.031 (-0.004- 0.065) | 0.018 | 0.081 |
|  | GT3 | 5 | 0.027 (0.001- 0.052) | 0.013 | 0.041 |
|  |  |  |  |  |  |
| Exposure | Intercept |  | 0.019 (0.008- 0.031) | 0.006 | < 0.001 |
|  | SOF-VEL-VOX 12 | 5 | reference | reference | reference |
|  | Matched placebo 12 | 1 | 0.027 (-0.009- 0.062) | 0.018 | 0.139 |
|  | SOF-VEL-VOX 4 | 1 | 0.012 (-0.074- 0.098) | 0.044 | 0.787 |
|  | SOF-VEL-VOX 6 | 5 | 0.001 (-0.027- 0.029) | 0.014 | 0.965 |
|  | SOF-VEL-VOX 8 | 8 | -0.002 (-0.017- 0.013) | 0.008 | 0.832 |
|  | SOF-VEL-VOX+RBV 12 | 1 | 0.021 (-0.057- 0.098) | 0.04 | 0.603 |
|  | SOF-VEL-VOX+RBV 8 | 1 | 0.013 (-0.05- 0.076) | 0.032 | 0.69 |
|  | SOF-VEL 12 | 3 | -0.01 (-0.024- 0.003) | 0.007 | 0.145 |

CI, confidence interval; SE, standard error

1. Comparing risk ratio

| Covariate | | Studies number | Event | Total | RR (95% CI) | P-value | IRR (95% CI) |
| --- | --- | --- | --- | --- | --- | --- | --- |
| Subgroup-analysis |  |  |  |  |  |  |  |
|  | Cirrhosis(-) | 5 | 0 | 131 | 0.016 (−0.005, 0.038) | 0.131 | Reference |
|  | Cirrhosis(+) | 9 | 17 | 384 | 0.034 (0.016, 0.052) | <0.001 | 12.000 (0.727, 198.160) |
|  | Cirrhosis(±) | 11 | 32 | 1800 | 0.013 (0.008, 0.018) | <0.001 | 4.764 (0.293, 77.366) |
| Grade 3 events |  |  |  |  |  |  |  |
|  | Hyperglycemia | 25 | 49 | 2315 | 0.015 (0.010, 0.020) | < 0.001 | Reference |
|  | Hemoglobin | 16 | 28 | 2154 | 0.009 (0.005, 0.013) | < 0.001 | 0.614 (0.387, 0.973) |
|  | Lymphocyte count | 10 | 13 | 1957 | 0.004 (0.001, 0.006) | 0.007 | 0.314 (0.171, 0.577) |
|  | Neutrophil count | 17 | 11 | 2118 | 0.005 (0.002, 0.008) | 0.002 | 0.245 (0.128, 0.471) |
|  | Platelet count | 25 | 21 | 2315 | 0.007 (0.004, 0.011) | < 0.001 | 0.429 (0.258, 0.712) |
|  | ALT | 16 | 7 | 2154 | 0.002 (0.000, 0.004) | 0.029 | 0.154 (0.07, 0.338) |
|  | AST | 17 | 14 | 2069 | 0.003 (0.000, 0.005) | 0.021 | 0.32 (0.177, 0.577) |
|  | Lipase | 19 | 33 | 2118 | 0.011 (0.007, 0.016) | < 0.001 | 0.736 (0.475, 1.14) |
|  | Total bilirubin | 13 | 6 | 2072 | 0.002 (0.000, 0.004) | 0.035 | 0.137 (0.059, 0.319) |
| Risk factors |  |  |  |  |  |  |  |
|  | HCV GT3 C(+) | 3 | 11 | 147 | 0.064 (0.017, 0.111) | 0.007 | Reference |
|  | Cirrhosis(+) | 9 | 17 | 384 | 0.034 (0.016, 0.052) | <0.001 | 0.592 (0.284, 1.233) |
|  | HCV GT3 | 5 | 15 | 260 | 0.048 (0.014, 0.082) | 0.006 | 0.771 (0.364, 1.634) |
| CI, confidence interval; SE, standard error; RR, risk ratio; IRR, incidence risk ratio | | | | | | | |

1. Certainty of evidence

| **SOF/VEL/VOX compared to Placebo for the impact on serum hyperglycemia** | | | | | |
| --- | --- | --- | --- | --- | --- |
| **Patient or population:** The impact on serum hyperglycemia  **Setting:** The impact of Sofosbuvir/Velpatasvir/Voxilaprevir treatment on serum hyperglycemia in HCV infections  **Intervention:** SOF/VEL/VOX  **Comparison:** Placebo | | | | | |
| **Outcomes** | **№ of participants (studies) Follow-up** | **Certainty of the evidence (GRADE)** | **Relative effect (95% CI)** | **Anticipated absolute effects** | |
|  |  |  |  | **Risk with Placebo** | **Risk difference with SOF/VEL/VOX** |
| Grade 3 hyperglycemia | 2315 (5 RCTs) | ⨁◯◯◯ Very low^a,b^ | **RR 1.07** (0.57 to 2.01) | 20 per 1,000 | **1 more per 1,000** (9 fewer to 20 more) |
| ***The risk in the intervention group** (and its 95% confidence interval) is based on the assumed risk in the comparison group and the **relative effect** of the intervention (and its 95% CI). **CI:** confidence interval; **RR:** risk ratio | | | | | |
| **GRADE Working Group grades of evidence** **High certainty:** we are very confident that the true effect lies close to that of the estimate of the effect. **Moderate certainty:** we are moderately confident in the effect estimate: the true effect is likely to be close to the estimate of the effect, but there is a possibility that it is substantially different. **Low certainty:** our confidence in the effect estimate is limited: the true effect may be substantially different from the estimate of the effect. **Very low certainty:** we have very little confidence in the effect estimate: the true effect is likely to be substantially different from the estimate of effect. | | | | | |

**Explanations**

a. All studies were open-label trials

b. the event rate very low
